# Risk Factors Associated with Peer Victimization and Bystander Behaviors among Adolescent Students

**DOI:** 10.3390/ijerph13080759

**Published:** 2016-07-27

**Authors:** Zepeng Huang, Zhenni Liu, Xiangxiang Liu, Laiwen Lv, Yan Zhang, Limin Ou, Liping Li

**Affiliations:** 1 Injury Prevention Research Center, Shantou University Medical College, 22 Xin Ling Road, Shantou 515041, China; idenge@foxmail.com (Z.H.); xxliu2014@126.com (X.L.); laiwen@tropmedres.ac (L.L.); 2 Fu Jian Province Center for Disease Control and Prevention, 76 Jin Tai Road, Fu Zhou 350001, China; liuzhennijy@163.com; 3 The Second People’s Hospital of Long Gang District, 175 Ji Hua Road, Shenzhen 518172, China; zyfish525@126.com; 4 The First Affiliated Hospital of Shantou University Medical College, 57 Chang Ping Road, Shantou 515041, China; oulimin138@sina.com

**Keywords:** bullying, adolescents, risk factors, bystander

## Abstract

Despite the prevalence of the phenomena of peer victimization and bystander behaviors, little data has generated to describe their relationships and risk factors. In this paper, a self-administered survey using a cross-sectional cluster-random sampling method in a sample of 5450 participants (2734 girls and 2716 boys) between 4th and 11th grades was conducted at six schools (two primary schools and four middle schools) located in Shantou, China. Self-reported peer victimization, bystander behaviors and information regarding parents’ risky behaviors and individual behavioral factors were collected. Multinomial logistic regression analysis was applied to evaluate risk factors affecting peer victimization and bystander behaviors. The results indicated that urban participants were more likely to become bullying victims but less likely to become passive bystanders. Contrarily, bullying victimization was related to the increasing of passive bystander behaviors. Father drinking and mother smoking as independent factors were risk factors for peer victimization. Participants who were smoking or drinking had a tendency to be involved in both peer victimization and passive bystander behaviors. This study suggested that bystander behaviors, victims’ and parents’ educations play a more important role in peer victimization than previously thought.

## 1. Introduction

Peer victimization is a popular public health topic all over the world from different perspectives, and the issue may vary in different areas and research settings. Peer victimization can lead to negative actions such as attacks, harm and intimidation, driven repeatedly by peers’ conscious, deliberate and aggressive intentions [1]. Many studies on peer victimization have been conducted in developed countries (e.g., USA, Europe and Canada); the prevalence of bullying victimization was between 10.6% and 81.0% [2,3,4,5,6]. However, few studies have been conducted in developing countries. In 2010, a worldwide health survey showed that the incidence of bullying victimization among 19 low-and middle-income countries ranged from 7.1% in Tajikistan to 60.9% in Zambia [7]. In Asia, the literature reports that about 20.0%–62.0% of school-aged students had been bullied by peers [8], with the lowest incidence corresponding to Guangdong (6.0%) and the highest to Beijing (20%) in mainland China [9,10,11,12].

Bullying victimization may lead to serious short-term and long-term adverse outcomes, especially psychosocial disorders (e.g., anxiety, loneliness, low self-efficacy) and dysfunctional behavior (e.g., delinquency) [13]. Many articles on victimization focus on the bully-victim diad or bullying itself, while few were aimed at bystanders, although bystander behavior is considered to be more prevalent than bullying victimization [14].

Bystanders, synonymous with “viewers”, “onlookers”, “passersby” and “witnesses”, are considered as “a person present but not involved” and can present in the form of assistants, helping bullies to bully victims, reinforcers, cheering and laughing to support bullies, outsiders, who pass by or watch to one side and defenders, supporting or helping victims [15,16]. Salmivalli [16] conducted a survey of Finnish grade six students that indicated that the percentages of “reinforcers”, “assistants”, “defenders” and “outsiders” were 19.5, 6.8, 17.3 and 23.7, respectively. Most studies on bystanders have shown that the majority of students had ever witnessed peer victimization [17,18], but chose not to give a hand to the victims if there was more than one person present [19]. There is increasing evidence showing that self-reported exposure to indirect violence, defined as witnessing or hearing violence, was related to behavioral dysfunction and mental maladjustment [20].

Given that peer victimization and bystander behaviors are a “group phenomenon” [21] both exposure to bullying behaviors and related to short- and long-term negative outcomes, addressing this pervasive problem and increasing the understanding of risk factors may help inhibit or stop bullying. Literature summarizes the following variables as associated with peer victimization and bystander behaviors:

(1) *Community characteristics*: The data collected to differentiate between peer victimization or bystander behaviors between urban and rural areas remains mixed and inconclusive. A study by Lowry [22] indicated that rural students were more likely to be bullied by peers. On the other hand, Rezapour [23] showed that there existed no significant differences in prevalence of peer victimization between urban areas and rural areas. A review by Buka [24] manifested that urban residents were more likely to witness violence. Observational research from Sherer [25] illustrated that no significant difference between witnessing violence was found between rural and inner-city areas;
(2) *Socio-demographic information*: Gender is a predictor of peer victimization and bystanders; many studies have indicated that boys were more likely to be bullied and to be passive bystanders than girls [16,17,26,27,28]. However, some scholars have argued that girls were more related to indirect bullying behaviors (verbal bullying and social manipulation) [6]. Students were less likely to be exposed to bullying victimization with increasing age/grade [29,30,31]. Data collected at two Toronto (Canada) elementary schools showed that younger students were more apt to support victims [17]. In an observational research on Grade 4 to 11 students in Canada, the respondents reported that passive bystanders’ behaviors aggrandized with increasing grade level [28];
(3) *Parents’ risk behaviors*: There is little data documenting an association between parents’ smoking/drinking behaviors and bullying victimization/bystander behaviors. Routine activities theory described that children would be far away from potential aggressors or be target as victims when they were more connected with and attached to the well-being of their parents. Conversely, parents’ smoking/drinking behaviors tended to present an obstacle to proper care and supervision [32]. Children who lack care and supervision have a trend to become victims or passive bystanders [33]. There is evidence of a link between parents’ smoking/drinking behaviors and bullying victimization [32,34], hence, we supposed that parents’ smoking/drinking behaviors were connected with children’s bullying victimization and bystander behaviors;
(4) *Behavioral characteristics*: The relationship between smoking/drinking behaviors and bullying victimization/bystander behaviors was mixed. Many studies have illustrated that children’s own smoking/drinking behaviors are risk factors to bullying victimization/bystander behaviors [6,25,35], whereas some scholars have suggested that smoking/drinking behaviors were not predictors of bullying victimization/bystander behaviors [36].

Factors influencing peer victimization and bystander behavior have been investigated in previous studies [20], but little data was generated to explore the relationship between peer victimization and bystander behaviors. Pathological reaction patterns [37], describe how each role could shift to another propelled by counter-identification, projective identification and splitting. The relationship of bullying victimization and bystander behaviors could be analogous to the mass-law equation in chemistry whereby one side of the equation could transform into the other by dynamic conditions which affect the components. The dynamic conditions were likely to be some elements influencing the two sides’ components, such as psychological changes or emotional responses. In clinical experience, bystanders with feelings of transfixion, helplessness and impotence (dynamic conditions) had a trend to later be victims themselves. Oppositely, victims may switch to be bystanders because of psychiatric illness (dynamic conditions). Moreover, children bystanders could not deal with bullying behaviors well so that he/she would be bullied as a wimp [15]. Empirical research conducted in Taiwan manifested that fatalism mediated the impact on past victimization on defender and outsider behaviors [38]. Reanalyzing almost 20 year old data, Zimmerman [20] found that higher rates of personal victimization were associated with higher risks to witness violence. In contrast, youths with higher rates of witnessing violence were not linked to higher risks of being personally victimized. With the mixed results above and cultural variance, in this study, we put forward the following questions:

First of all, were peer victimization or bystander behaviors different between the urban and rural areas? We hypothesized that peer victimization was more likely to take place in rural areas, while passive bystander behaviors were more likely to happen in urban areas.

Secondly, what were the risk factors predicting peer victimization or bystander behaviors? We hypothesized that boys, younger students, father smoking, father drinking, mother smoking, mother drinking, smoking and drinking were risk factors to peer victimization, while boys, elder students, father smoking, father drinking, mother smoking and mother drinking raised the risks of passive bystander behavior.

Finally, were peer victimization and bystander behaviors increased risks for each other? We hypothesized that peer victimization was a risk factor of passive bystander behaviors and vice versa. If this hypothesis were correct, prevention of bullying victimization should not only focus on bullying itself, but also target bystanders.

## 2. Methods

### 2.1. Ethics Statement

All subjects gave their informed consent for inclusion before they participated in the study. The study was conducted in accordance with the Declaration of Helsinki, and the protocol was approved by the Ethics Committee of the Medical College of Shantou University (Code: SUMC2013XM-0096).

### 2.2. Participants and Sampling

A cross-sectional cluster-random survey was conducted among six schools (two primary schools and four middle schools) located in Shantou, in East Guangdong, from 17 June to 29 October 2013. Participants below 4th grades were removed due to perceived cognitive deficiency and those exceeding 11th grades were excluded due to their heavy academic workload.

Before the survey, the purpose of the study was communicated and potential assistance for the study was sought from the principals of the chosen schools through a telephone request. All principals agreed to participate in the survey. Subsequently, the investigation was conducted during regular class time with the help of instructors. Investigators informed the participants about the survey purpose, significance, completion methods and precautions in accordance with uniform methods and standards. Students could consult at any time if they had any doubts about the investigation. In total, 5841 questionnaires were collected, among which, 391 questionnaires were ruled out as a result of a large percentage of missing data. In addition, three missing values were filled after logic inspection and eight data errors were fixed after telephone confirmation. The total number of valid questionnaires was thus 5450, resulting a response rate of 93.3%.

### 2.3. Measurement

The questionnaire was consisted of six sections: (1) community characteristics: rural and urban school, identified by their administrative planning locations, which indicated that the rural area is a place where the laborers engaged in agricultural production and urban area is a larger settlement dedicated to non-agricultural industry and with a non-agricultural population; (2) socio-demographic information, including gender, age and grade (elementary (grades 4–6), junior (grades 7–9) and senior (grades 10–11)); (3) parents’ risk behaviors: father smoking, father drinking, mother smoking and mother drinking (Yes or No); (4) behavioral characteristics, including smoking and drinking (Yes or No); (5) bystander-related questions, seen in Table 1, in which situations in last six months were asked of participants. Bystander behaviors were classified into active bystander behaviors (dissuade; report to teachers or school officials or call the police) and passive bystander behaviors (watch in fear or leave indifferent); (6) a Multidimensional Peer Victimization Scale (MPVS) [39,40], with 16 items, including four dimensions: physical victimization, verbal victimization, social manipulation and attack on property, asked about bullying victimization in the last semester. Each item was estimated at a five-point Likert Scale by participating students, ranging from none, once a time, twice times, three times to more than three times. The reliability of the questionnaire was analyzed by Cronbach’s Alpha, whose coefficients for total victimization, physical victimization, verbal victimization, social manipulation and attack on property were 0.926, 0.881, 0.885, 0.786 and 0.801 respectively, indicating good internal consistency and reliability. Peer victimization was defined as scores equal or greater than the mean standard deviation.

### 2.4. Statistical Analysis

After the data were input by Epidata 3.1 software (EpiData Association, Odense, Denmark), databases were established using Excel 2010 (Microsoft Corp., Redmond, WA, USA) and the Statistical Package for Social Science version 19.0 software (SPSS 19.0, SPSS Inc., Chicago, IL, USA) was adopted for data analysis. Descriptive statistics for related community characteristics, social-demographic, and behavior characteristics information and bystander behavior-related questions were presented. The chi-square test was adopted to compare the differences of peer victimization in community characteristics, gender, grade and bystander behaviors and the difference of bystander behaviors in community characteristics, gender, grade and peer victimization. Finally, excluding the variable-age (we deemed grade as a stronger predicted factor), multinomial logistic regression analysis was carried out to explore risk factors between peer victimization and bystander behaviors. The including and excluding standard in the equation were 0.05 and 0.10, respectively.

## 3. Results

### 3.1. The Information of Social and Behavior Characteristics

Of total 5450 participants, there were 1344 (24.66%) rural and 4106 (75.34%) urban students, including 2734 (50.17%) girls and 2716 (49.83%) boys. The age of the selected students ranged from 10 to 19 with a mean age of 15 (14.49 ± 2.07). The majority of selected samples had never smoked (96.64%) but had drunk (63.34%). Among all participants, 1521 (27.91%) students had ever witnessed school bullying in the last six months, among which 8.31% of cases took place inside the school, 7.60% happened around the school gate and 12.00% outside the school. Among these bullying events, 73.37% cases involved only students, 15.84% took place between students and social youths, 0.85% cases were between students and teachers and 9.93% were others. When asked how they would deal with school bullying if faced, 4101 (75.25%) students preferred to be active bystanders, including 9.16% dissuaded, 59.16% reported to teachers or school officials and 6.94% called police, while passive bystanders accounted for 24.75%, including 6.64% watched in fear, 18.11% left indifferent. Detailed data are presented in Table 2.

### 3.2. Differences of Peer Victimization and Bystander Behaviors in Community Characteristics, Gender and among Grades

As shown in Table 3, there were 807 (14.81%) bullying victims and 1349 (24.75%) passive bystanders. Respondents self-reported that bullying victimization were more likely to take place in the urban area, while more passive bystander behaviors happened in the rural area (OR = 1.23, 95% CI = 1.02–1.47; OR = 0.77, 95% CI = 0.67–0.89, respectively). Significant gender differences between peer victimization and passive bystander behaviors were found, which indicated that boys were more likely to involve in peer victimization and passive bystander behaviors (OR = 1.64, 95% CI = 1.42–1.91, OR = 1.46, 95% CI = 1.29–1.65). With the increase in grades, bullying victimization declined (21.5% vs. 17.3% vs. 10.1%), while passive bystander behaviors augmented (18.3% vs. 25.4% vs. 26.8%). Prevalence of peer victimization and passive bystander behaviors were significantly different between grades.

### 3.3. The Relationship between Peer Victimization and Passive Bystander Behaviors in Univariate Analysis

Table 3 also revealed the relationship between peer victimization and passive bystander behaviors by using univariate analysis. Of 5450 participants, 270 (20.01%) passive bystanders were considered as bullying victims, while conversely, 33.46% peer victims were regarded as passive bystanders. A significant difference was found between peer victimization and passive bystander behaviors (OR = 1.66, 95% CI = 1.41–1.95).

### 3.4. Results of Multivariate Logistic Regression toward Peer Victimization and Passive Bystander Behaviors

Controlling of all the other factors, multivariate logistic regression analysis was conducted to seek factors related to peer victimization and bystander behaviors. The result indicated that urban (vs. rural) (OR = 1.82, 95% CI = 1.48–2.23), boys (OR = 1.38, 95% CI = 1.18–1.62), father drinking (OR = 1.20, 95% CI = 1.01–1.42), mother smoking (OR = 2.26, 95% CI = 1.48–3.46), smoking (OR = 1.82, 95% CI = 1.30–2.67), drinking (OR = 1.49, 95% CI = 1.24–1.79) and passive bystander behaviors (OR = 1.64, 95% CI = 1.39–1.94) were raised risks for bullying victimization, while junior (OR = 0.47, 95% CI = 0.38–0.58) and senior (OR = 0.23, 95% CI = 0.18–0.29) grade were protective factors. Predictors of passive bystander behaviors included urban (vs. rural) (OR = 0.57, 95% CI = 0.48–0.66), gender (OR = 1.34, 95% CI = 1.18–1.53), grade (junior OR = 1.61, 95% CI = 1.30–1.99; senior OR = 1.83, 95% CI = 1.48–2.28), smoking (OR = 1.43, 95% CI = 1.20–1.70); drinking (OR = 1.63, 95% CI = 1.40–1.89) and peer victimization (OR = 1.64, 95% CI = 1.39–1.95). Details are shown in Table 2.

## 4. Discussion

By recruiting 5450 participants residing in Shantou City, the current study aimed to address the three following research questions: Were peer victimization and bystander behaviors different between urban and rural areas? What were the risk factors affecting peer victimization and passive bystander behaviors? Do peer victimization and passive bystander behaviors raise the risks for each other?

With regard to the first question, contradicting our hypothesis, the results revealed that more urban students were liable to be bullied as victims. One study with 834 middle school samples in Iran showed that adolescents from rural areas were more likely to perform verbal bullying victimization [23]. Evidence from Paul indicated that no significant difference of bullying victimization was found between urban and rural public schools, while scores of peer victimization were higher in rural areas [41]. Literature from the WHO even showed that urban upgrading was likely to reduce bullying incidences [42]. These contradictions may be due to different educational resources and the cognition of bullying victimization [43]. Besides, the different definitions of urban or rural areas were expected to result in the occurrence of such variation. Some studies defined urban/rural areas by their school locations, which was consistent with the definitions used in this study, while others did it through residence areas [23]. Further studies should be conducted to explore related factors. For example, generally, urban areas are superior in economic development, which reduces the possibility of bullying behaviors [42]. However, this study had difficulty in providing data to verify economic differences between the urban and rural areas in our samples. Different from researches conducted in Thailand and America [25,44], rural youths were found to be more likely to be passive bystanders when facing bullying behaviors, which contributed to the variety of definitions, measures and cultures too.

In terms of the second question, in line with past studies [13,17], gender was a risk factor for both peer victimization and passive bystander behaviors. Girls, owing to their social roles—with higher level of empathy and sensitivity—were more likely to be active bystanders [16]. However, some scholars have argued that there are no significant differences between peer victimization and bystander behaviors by gender [11,45], which could result in their increased involvement in indirect victimization (e.g., verbal victimization) due to girls’ introversion and effeminacy. Consistent with a previous study [13], higher grade students were less exposed to bullying victimization because of the higher study stress, stronger physical conditions and mind-set maturity. In line with our hypothesis, higher grade students were more apt to be involved in passive bystander behaviors. This finding was similar to previous studies pointing out that younger students were more likely to help victims when they witnessed violence [17]. Trach also reported that younger students (i.e., grades 4–5) were more engaged in directly intervention, such as talking to an adult, telling the bully to stop, or helping the victims [28]. What made it unclear was whether the observed differences reflect a change in age-related behaviors within the individual. In line with past literatures [32,34], father drinking and mother smoking were associated with peer victimization. Parents involved in cigarette or alcohol use may be less likely to supervise their children’s behaviors, which was consistent with the routine activities theory that the lack of parental supervision was related to children’s victimization [32]. Conversely, a study based on a sample of 1344 fourth-grade elementary school students indicated that no significant differences were found between parents’ alcohol problems and bullying victimization [46]. These differences may be a result of differences in concepts, samples, countries and cultures. Children’s self-reported smoking or drinking behaviors were associated with both peer victimization and passive bystander behaviors. Past studies also supported that adolescents involved in smoking or drinking behaviors were more likely to witness violence or be bullied as victims [25,47]. Possible explanations may be that children who smoked or drank became undesirable and therefore had a tendency to be victims, or as passive bystanders due to anxiety about retaliation [15]. However, owing to the natural deficiencies of a cross-sectional survey, we could not determine the true temporal order. The reverse explanation could be that victim and bystander behaviors may reflect development [36]. Further conclusions will require a cohort study to verify their causality.

With respect to the third question, this study showed that passive bystander behavior was related to an increasing in peer victimization. Meanwhile, peer victims were more likely to be involved in passive bystander behaviors, which was partly in line with previous studies. Research conducted in Chicago illustrated that personal bullying victims were more likely to witness violence, while violence witnessing did not predict personal victimization [20]. Literature in Taiwan showed that direct victimization and bystander behaviors were related in univariable analysis, whereas these connections disappeared in multivariable analysis [48]. A possible explanation could be the differences in measures and statistical methods. Although this paper couldn’t verify the pathological reaction patterns directly, there were evidences showing that peer victimization and bystander behaviors were both in keeping with post-traumatic stress disorder (PTSD) (dynamic conditions) [24,26]. All these hinted at the correctness of the model. More variables will be analyzed to explore related mechanisms between peer victimization and bystander behaviors in follow-up research. The possible interchange with peer victimization and bystander behaviors also hinted that the absence of bystander education could result in bullying behaviors in China. Literature about bystander prevention was effective at reducing bullying behaviors [49], which indicated that bystanders were more likely to stop bullying behaviors when they had responsibility to intervene, felt confidence in their skills and had faith in the value of intervening beyond the costs. Further studies should be focused on identifying types of bystander’s behaviors, encouraging students to take positive measures in the face of bullying incidents and teaching them related skills to deal with it smartly.

There were some limitations of this study. Firstly, causal relationships could not be proved for the cross-sectional study. What this study could clarify was only the relationship between dependent variables and independent variables. For example, the relationship between peer victimization and bystander behavior was temporary (whether bullying victimization was a herald or a consequence of passive bystander behaviors was unknown), which precluded us from determining the temporal order of the associations. Further longitudinal studies should be performed to verify the connections between variables and bullying behaviors. Secondly, selection bias could not be resolved because the reasons (i.e., dropping out, truancy, disability, etc.) for the absence of students from their schools were not determined. Evidence had proved that students who had ever skipped class were likely to be bullied [50]. In addition, this study did not cover students below 4th grade or above 11th grade, so it could not describe the complete status of bullying victimization or bystander behaviors among elementary and middle schools. Further research will be hopefully be conducted to fill this gap. Self-report forms and real name were displayed. Students may not report bullying or bystander behavior for fear of suffering bullying again if peer bullies thought they might be reported. Nevertheless, it was acceptable to adopt self-report forms to investigate the topic [51]. Moreover, this study did not paid close attention to cyber-victimization, which may do more harm to adolescents [52,53,54]. Thirdly, as a results of the data of this study were collected based on participants’ recall about past events, whereby participants were requested to recall their peer victimization states during the last semester and bystander behaviors in the last six month, recall bias was difficult to avoid. Measurement instruments of bystander behaviors were self-designed, resulting in difficulty to identify other roles of special types of bystanders, such as assistants and reinforcers, who play key roles in the bullying process and its consequences [16,38]. Further studies will adopt a well utilized measurement instrument to assess and compare bystander behaviors against a Chinese cultural background. Despite the limitations mentioned above, this study still offered crucial data to explore the risk factors for peer victimization and bystander behaviors. The results firstly displayed that peer victimization and passive bystander behaviors were risk factors associated with each other, by analyzing the quantitative data. We expect more repeatable data can be extended to other countries.

## 5. Conclusions

In conclusion, peer victimization was related to an increase in passive bystander behaviors, and vice versa, which indicated that peer victimization should be taken into account as a group phenomenon. This study suggested that active prevention and control measures should not only take into account the bullying itself, but need to focus on community characteristics, individual behaviors and parents’ risk behaviors. Education directed toward bullying victimization were sometime centered on bullies or victims in ways which always tracked back to the change of attitude, behaviors and mental states [55]. However, this study put forward an important clue that education directed towards parents was likely to reduce bullying victimization too. When it came to implications of practice, interventions on peer victimization should address the whole group rather than individual bullies or victims [16]. Bystanders were encouraged to be more active in defending and supporting victims when the witnessed bullying [21,31]. In view of the results of this study, if students could actively deal with bullying incidents when witnessed, the probability of becoming bullying victims would decline. There was evidence that active bystander behaviors, more in line with healthy psychosocial functioning (e.g., with higher level of empathy, self-esteem and self-efficacy), could reduce the degree of bullying as well as its consequences [21]. Further studies should investigate the roles of bystander behaviors and related mechanisms in preventing bullying victimization.

## Figures and Tables

**Table 1:** Bystander behavior-related questions.

Questions	Options
Do you have ever witnessed school bullying behaviors?	A Yes; B No
Where do you witness bullying accidents?	A Inside the school; B In the school gate; C Outside the school
Who are the participants in bullying accidents you saw?	A Classmates; B Schoolmates; C Schoolmates and off-campus students; D Students and unemployed youth; E Students and teachers; F Others
What will you do when witness school bullying behaviors?	A Dissuade; B Report to teachers or school officials; C Call the police; D Watch in fear; E Leave indifferent

**Table 2:** Shared and independent factors of peer victimization and passive bystander behaviors.

Variables	N	Peer Victimization		Passive Bystander Behaviors	
		OR	95% CI	OR	95% CI
Urban (vs. rural)	4016	1.82 ***	1.48–2.23	0.57 ***	0.48–0.66
Gender	2716	1.38 ***	1.18–1.62	1.34 ***	1.18–1.53
Junior (vs. elementary)	2015	0.47 ***	0.38–0.58	1.61 ***	1.30–1.99
Senior (vs. elementary)	2451	0.23 ***	0.18–0.29	1.83 ***	1.48–2.28
Father smoking	3803	0.96	0.79–1.12	0.93	0.81–1.08
Father drinking	3185	1.20 *	1.01–1.42	1.13	0.99–1.30
Mother smoking	121	2.26 ***	1.48–3.46	0.87	0.57–1.35
Mother drinking	622	1.19	0.94–1.50	1.21	1.00–1.48
Smoking	758	1.82 ***	1.48–2.24	1.43 ***	1.20–1.70
Drinking	3452	1.49 ***	1.24–1.79	1.63 ***	1.40–1.89
Passive bystander behaviors	1349	1.64 ***	1.39–1.94	NA	NA
Peer victimization	807	NA	NA	1.64 ***	1.39–1.95

Notes:* *p* < 0.05, *** *p* < 0.001.

**Table 3:** The differences between peer victimization and bystander behaviors according to community characteristics, gender and grade.

Variables	Type	N	Peer Victimization			Passive Bystander Behaviors		
			%	OR (95% CI)	*p*	%	OR (95% CI)	*p*
Community characteristics	Rural	1344	12.95	1		28.5	1	<0.01
	Urban	4106	15.42	1.23 (1.02–1.47)	0.03	23.53	0.77 (0.67–0.89)	
Gender	Girls	2734	11.70	1		21.29	1	<0.01
	Boys	2716	17.93	1.64 (1.42–1.91)	<0.01	28.24	1.46 (1.29–1.65)	
Grade	Elementary	984	21.5	1	<0.01	18.3	1	<0.01
	Junior	2015	17.3	0.76 (0.63–0.92)		25.4	1.52 (1.26–1.84)	
	Senior	2451	10.1	0.41 (0.33–0.50)		26.8	1.64 (1.36–1.97)	
Passive bystander behaviors	No	4101	13.09	1	<0.01	NA	NA	NA
	Yes	1349	20.01	1.66 (1.41–1.95)		NA	NA	NA
Peer victimization	No	4643	NA	NA	NA	23.24	1	<0.01
	Yes	807	NA	NA	NA	33.46	1.66 (1.41–1.95)	

Notes: % is the percent of peer victimization or passive bystanders. *p* value is examined by chi-square test.
